# Comparative investigation of extended-gate GaAsSb/InGaAs HTFET biosensors for label-free biomolecule detection

**DOI:** 10.1186/s11671-026-04978-9

**Published:** 2026-10-15

**Authors:** Mahin Anjum, Md. Mehedi Hasan, Md. Feroz Ali, Md Shafiul Alam, Obaidullah Obaidi

**Affiliations:** 1 Department of Electrical and Electronic Engineering, Bangladesh Army University of Science and Technology, Saidpur, 5310 Nilphamari Bangladesh; 2 Department of Electrical and Electronic Engineering, Pabna University of Science and Technology, Pabna, 6600 Bangladesh; 3 Department of Electrical Engineering, College of Engineering, King Faisal University, Al Ahsa, 31982 Saudi Arabia; 4 Department of Energy Engineering, Faculty of Engineering, Kabul University, Kabul, 1006 Afghanistan

**Keywords:** TFET, MOSFET, Nanocavity, Biomolecules, Dielectric-modulation, Heterostructure, Sensitivity, Selectivity

## Abstract

The dielectric-modulated heterostructure tunnel FET (HTFET)-based nano-cavity integrated biosensors are emerging as energy-efficient, ultra-sensitive label-free detection platforms. This study investigates and compares three GaAsSb/InGaAs-based biosensor architectures: single cavity dual material double composite gate HTFET (SC-DM-DG HTFET), dual cavity dual material double composite gate HTFET (DC-DM-DG HTFET), and single cavity dual material extended double composite gate HTFET (SC-DM-EDG HTFET). The III–V heterostructure in the SC-DM-EDG HTFET enhances tunnelling probability, gate control, and on-current (*I*_*ON*_), thereby providing higher sensitivity than previously reported GaAsSb/InGaAs-based HTFET and FET biosensors. Sensitivity and selectivity parameters for *I*_*ON*_, transconductance (*g*_*m*_), and threshold voltage (*V*_*th*_) were evaluated for various neutral and charged biomolecules. Under the standard bias condition (*V*_DS_ = 1.5 V, *V*_GS_ = 1.3 V), the SC-DM-EDG HTFET exhibits the highest *I*_*ON*_ of 3.02 × 10^−4^ A/*μ*m among the three structures; under a separately re-optimized bias (*V*_DS_ = 0.9 V, *V*_GS_ = 1 V), the same structure instead achieves maximum sensitivities of 3.35 × 10^6^ (*I*_*ON*_) and 3.49 × 10^5^ (*g*_*m*_) for *K* = 12, at a correspondingly reduced *I*_*ON*_ of 1.91 × 10^−4^ A/*μ*m. The maximum *V*_*th*_ sensitivity of 0.49 is achieved by this structure under the standard bias condition. In biomolecule differentiation, the SC-DM-DG HTFET demonstrates peak *I*_*ON*_ and *g*_*m*_ selectivity values of 9.55 and 4.64 for *K* = 12, respectively, while the peak *V*_*th*_ selectivity value of 0.3 appears in the SC-DM-EDG HTFET also for the same biomolecule. Furthermore, the SC-DM-EDG HTFET achieves superior *I*_*ON*_, *g*_*m*_, and *V*_*th*_ sensitivities for charged biomolecules, confirming its robustness, selectivity, and potential for next-generation, high-performance biosensing applications.

## Introduction

The precise identification of nano-biomolecules has become essential with the rapid progress of microbiology. Biomolecules such as DNA, biotin, streptavidin, amino acids, APTES, gelatin, and various bacteria or viruses must be detected efficiently to understand abnormal cellular behavior [1, 2]. FET based biosensors have emerged as promising alternatives to conventional laboratory sensors because of their miniaturization, robustness and low power consumption [1, 3].

Among these, dielectrically modulated MOSFET biosensors with nanocavities have gained prominence for their flexibility and accuracy [4]. A functionalized nano-gap dielectric beneath the gate metal hosts selective receptors that capture target biomolecules, producing a gating effect translated into measurable outputs such as drain-current variation or threshold-voltage shift. Because different biomolecules exhibit distinct dielectric constants—streptavidin (*K* = 2.1), biotin (*K* = 2.63), gluten (*K* = 5), zenin (*K* = 7), keratin (*K* = 11), and gelatin (*K* = 12) [5, 6], their selective detection becomes feasible. However, despite their promise, MOSFET-based biosensors face intrinsic limits: short-channel effects (SCEs) and a 60 mV/dec subthreshold swing (SS) bound at room temperature [7, 8]. These restrict voltage scaling and sensing efficiency, while fabrication complexity and device variability hinder large-scale uniformity.

To address these constraints, TFETs have drawn attention for their steeper SS, stronger gate control, and higher sensitivity [9, 10]. TFETs operate via band-to-band tunnelling (BTBT) in a p-i-n or n-i-p configuration [11, 12], allowing sub-60 mV/dec switching and ultra-low-power operation. Yet early silicon-based homojunction TFETs delivered limited *I*_*ON*_ due to silicon’s indirect bandgap and high tunnelling mass. Heterojunction TFETs have emerged as a compelling solution to the intrinsic limitations of conventional TFETs by leveraging narrow-bandgap source materials to enhance band-to-band tunnelling through staggered or broken-gap alignment. However, IV–IV heterostructures, such as strained SiGe sources [13] and pocket-doped SiGe channels [14], remain constrained by lattice mismatch, limited band offsets, and low current drivability. This has accelerated the transition toward III–V systems, including InGaAs/Si [5], GaAs/AlGaAs/GaSb [15], and InAs/Si [16], which offer improved tunnelling efficiency but still face trade-offs between performance and manufacturability.

Among these, the *GaAsSb*/*InGaAs* material system stands out due to its compositionally tunable type-II alignment, enabling near broken-gap conditions and superior tunnelling characteristics. Notably, [17] experimentally fabricated *GaAsSb*/*InGaAs* quantum-well TFETs, establishing strong technological viability. Further validation is provided by [18], which demonstrated heterojunction Esaki diodes with excellent agreement between simulated and measured I–V characteristics, and by [19], which reported a *GaAsSb*/*InGaAs* heterojunction TFET with a nanoscale In_0.7_Ga_0.3_As pocket layer, achieving a high on-state current of 11.98 *μ*A/*μ*m at room temperature. Additional designs, including GAA and L-shaped TFETs [20, 21], further exploit broken or quasi-broken gap alignment to achieve high *I*_*ON*_ and steep SS.

Collectively, these studies confirm that *GaAsSb*/*InGaAs* heterojunction TFETs are technologically viable; however, the design space of such devices for biosensing remains insufficiently understood. In the present work, we comparatively investigate three *GaAsSb*/*InGaAs* HTFET biosensor topologies SC-DM-DG, DC-DM-DG, and SC-DM-EDG to determine how cavity placement, extended-gate engineering and bias condition influence the electrical and sensing characteristics. In contrast to earlier reports that mainly examined a single structure, this study provides a unified comparison of *I*_ON_, *g*_*m*_, and *V*_*th*_-based sensitivity and selectivity for both neutral and charged biomolecules, together with a physical interpretation based on electric-field modulation and surface-potential control. While prior biosensor studies such as [22, 23] demonstrated promising sensitivity using *GaAsSb*/*InGaAs*-based TFET configurations, they did not systematically address architecture-level trade-offs among current drivability, relative sensitivity, and extended-gate-assisted electrostatic control. This gap motivates the present comparative analysis, which aims to establish an architecture-guided *GaAsSb*/*InGaAs* biosensor design framework by revealing how cavity placement and extended-gate engineering govern electric-field enhancement, tunnelling behavior, sensitivity, selectivity, and current drivability. To the best of our knowledge, the extended dual-material-gate (EDG) architecture is reported here for the first time on a *GaAsSb*/*InGaAs* type-II heterojunction platform. In particular, by evaluating each architectural change at matched bias before separately re-optimizing the bias point, this work isolates which design element is actually responsible for the reported sensitivity improvement, showing that it is the extended-gate architecture acting jointly with bias re-optimization, rather than cavity placement or gate extension in isolation, that yields the highest sensitivity.

## Device structure and simulation framework

**Fig. 1:**
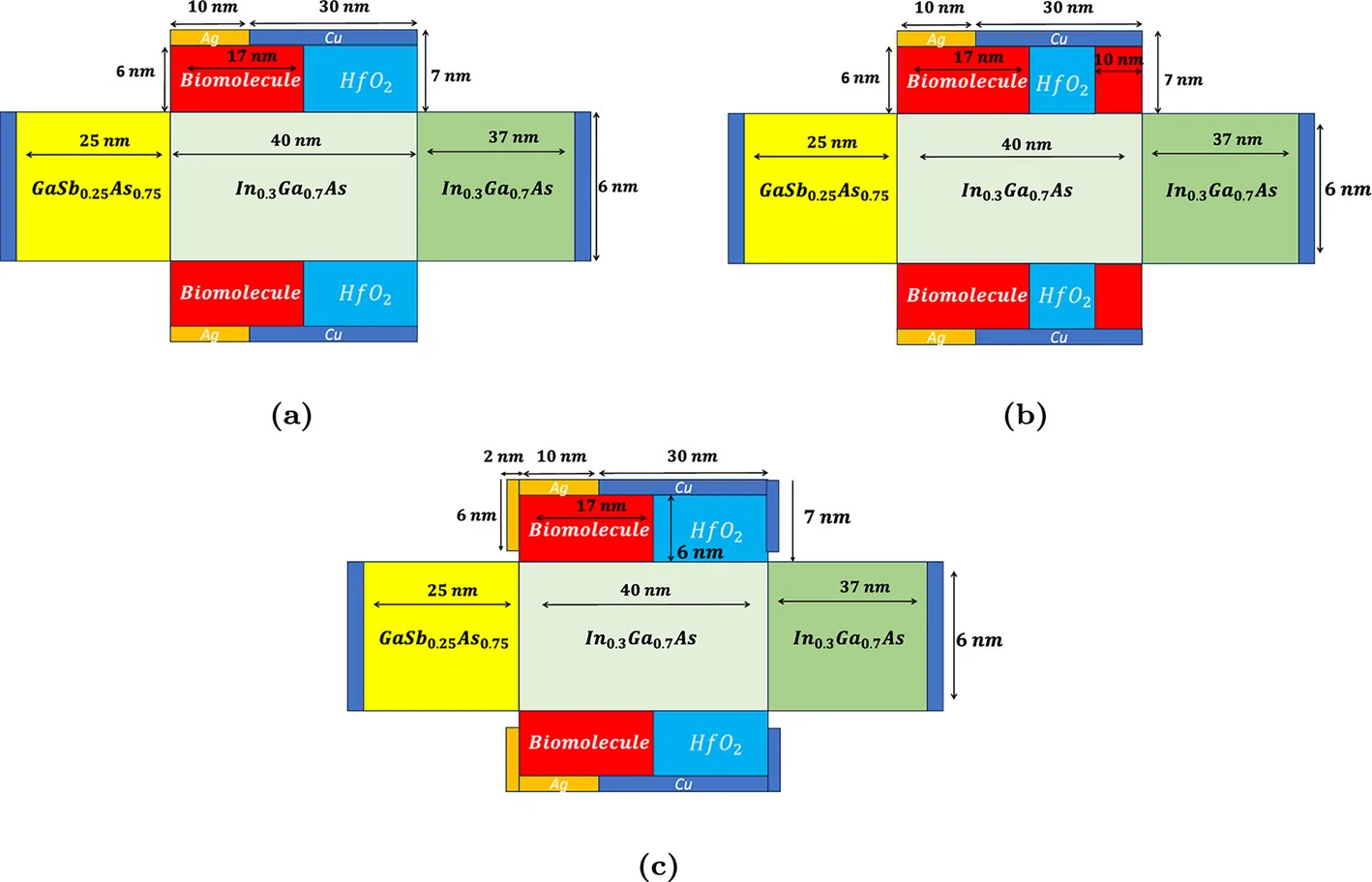
2D schematic diagrams: (**a**) SC-DM-DG HTFET (**b**) DC-DM-DG HTFET (**c**) SC-DM-EDG HTFET

The two-dimensional schematic structures along with dimensional parameters of the (a) SC-DM-DG HTFET, (b) DC-DM-DG HTFET, and (c) proposed SC-DM-EDG HTFET are illustrated in Fig. 1. *GaAsSb* is used as source material and *InGaAs* is being used as channel and drain material. Overall, it is a p-i-n structure. *InGaAs* is a low direct bandgap material, with very low electron tunnelling mass (*m*_*e*_) compared to silicon. This feature makes it suitable for channel region as tunnelling probability depends on bandgap and tunnelling effective mass. Again, *InGaAs* has electron and hole mobility many orders higher than that of silicon [24].

The compositions In_0.3_Ga_0.7_As and GaAs_0.75_Sb_0.25_ were chosen to form a moderate type-II GaAsSb/InGaAs heterojunction, thereby providing a balanced trade-off between tunnelling enhancement and interface quality. Based on Vegard-law interpolation, the lattice constants of In_0.3_Ga_0.7_As and GaAs_0.75_Sb_0.25_ are 5.7748Å and 5.7639Å, respectively, corresponding to a lattice mismatch of only  ~ 0.19%. This value is comparable to that of the widely used InP-matched In_0.53_Ga_0.47_As/GaAs_0.49_Sb_0.51_ system [18]. Although In-richer and Sb-richer alloy combinations may further lower the effective tunnelling barrier and enhance *I*_*ON*_, they also tend to produce a more aggressive staggered band alignment [20], which can compromise OFF-state controllability and increase the sensitivity of device performance to interface defects. Hence, the selected composition represents a compromise among low lattice mismatch, favorable type-II band alignment, threshold-voltage controllability, and sufficiently high band-to-band tunnelling (BTBT) probability for biosensing applications. Although the selected In_0.3_Ga_0.7_As/–GaAs_0.75_Sb_0.25_ heterojunction is not exactly lattice matched to a standard binary III–V substrate, its effective lattice constant ( ~ 5.769Å) lies closer to InP (5.8687Å) than to GaAs (5.65325Å). Therefore, an InP-based platform with a metamorphic or compositionally graded buffer is a realistic substrate route for epitaxial realization. Alternatively, a relaxed virtual substrate engineered near 5.769Å may be employed to further reduce mismatch-induced defects.

For all three structures, the source section is heavily doped with p-type dopants at a concentration of roughly 5 × 10^19^cm^−3^, guaranteeing a high carrier population required for efficient BTBT. The channel section carries a light n-type doping near 1 × 10^16^cm^−3^ to preserve low leakage current and to suppress the ambipolar response of the TFET. The drain section is given a moderate n-type doping of about 1 × 10^18^cm^−3^, ensuring that the series resistance stays sufficiently low to support large current conduction when the device operates in the on-state. Silver (Ag) and copper (Cu) serve as the gate and contact metals, with respective work functions of 4.2 eV and 4.7 eV. The gate lengths (*L*_gate1_, *L*_gate2_) are fixed at 10 nm and 30 nm. The higher work function of Ag at the tunnelling gate guarantees robust gate control over the tunnelling zone, whereas Cu delivers stable electrical connections for the auxiliary gate, source, and drain. The dual-gate TFET has dual benefits of higher *I*_ON_ and low off current (*I*_OFF_), thereby improving the overall $\frac{{I}_{{\rm{ON}}}}{{I}_{{\rm{OFF}}}}$ ratio. The gate metal work function is selected in a way that carrier tunnelling occurs exclusively when the nano-gap is occupied by biomolecules, thereby enabling a precise variation in the sensing parameter to be detected [16]. Moreover, the cavity height (*H*_gap_) is chosen to be 6 nm, a value comparable to those reported in [5, 25].

The SC-DM-DG HTFET represents the fundamental configuration, incorporating only a single cavity on each side of the gate. The cavity length (*L*_gap_) is defined through comprehensive simulations, and the optimal value is then chosen accordingly. The DC-DM-DG HTFET has an additional cavity on the drain side to achieve better control over dielectric modulation. The cavity dimensions are determined through detailed simulations, and the optimal cavity lengths (*L*_gap1_ = 17nm, *L*_gap2_ = 10nm) are subsequently selected. A dual-cavity design provides two sensing regions, so even small changes in dielectric constant produce a larger perturbation in the electric field. MOSFET operation relies on gate-channel coupling, which determines the parameters of the inversion layer in the channel. Thus, introducing a dual cavity can increase the sensitivity with respect to *K* dramatically. However, in TFETs, *I*_ON_ is very sensitive to the electrostatic potential near the tunnelling junction only, so introducing an extra cavity on the drain side should have very little impact on the sensitivities.

**Fig. 2:**
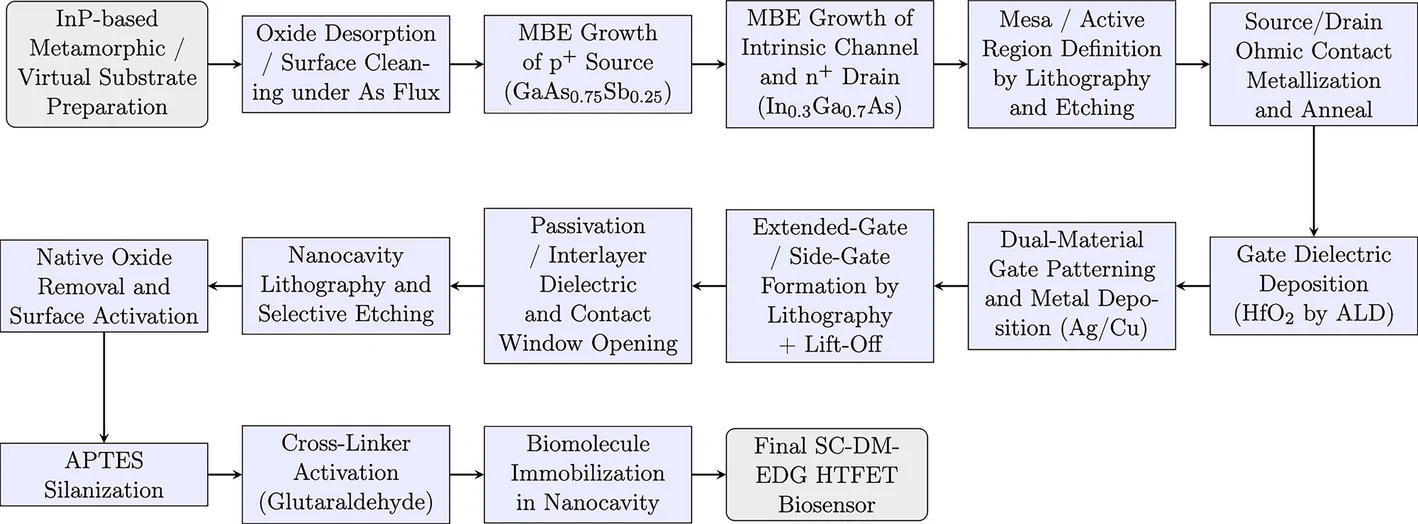
Proposed fabrication process flow for the SC-DM-EDG HTFET biosensor

The SC-DM-EDG HTFET is the most structurally complex among the three proposed devices; however, it offers several important advantages. The extended tunnelling gate improves electrostatic control near the source-channel tunnelling junction, thereby enhancing the local electric field and increasing the BTBT rate, while the extended auxiliary gate helps regulate thermionic emission [5]. Additionally, the extended tunnelling gate reduces leakage and ambipolar current [26]. The device incorporates two extended gates: an Ag-based extended gate on the tunnelling side and a Cu-based extended gate on the drain side. Owing to this configuration, the electric field at the source–channel junction is strengthened and the surface-potential profile becomes steeper, leading to improved current conduction and gate controllability. The geometric parameters of the extended gates, namely the thickness (*T*_EG_) and length (*L*_EG_), are illustrated in Fig. 1c. Although the fabrication procedure of the SC-DM-EDG HTFET can be relatively complex, a realistic overall process flow adapted from GaAsSb/InGaAs heterojunction diode fabrication [18] is shown in Fig. 2.

**Fig. 3:**
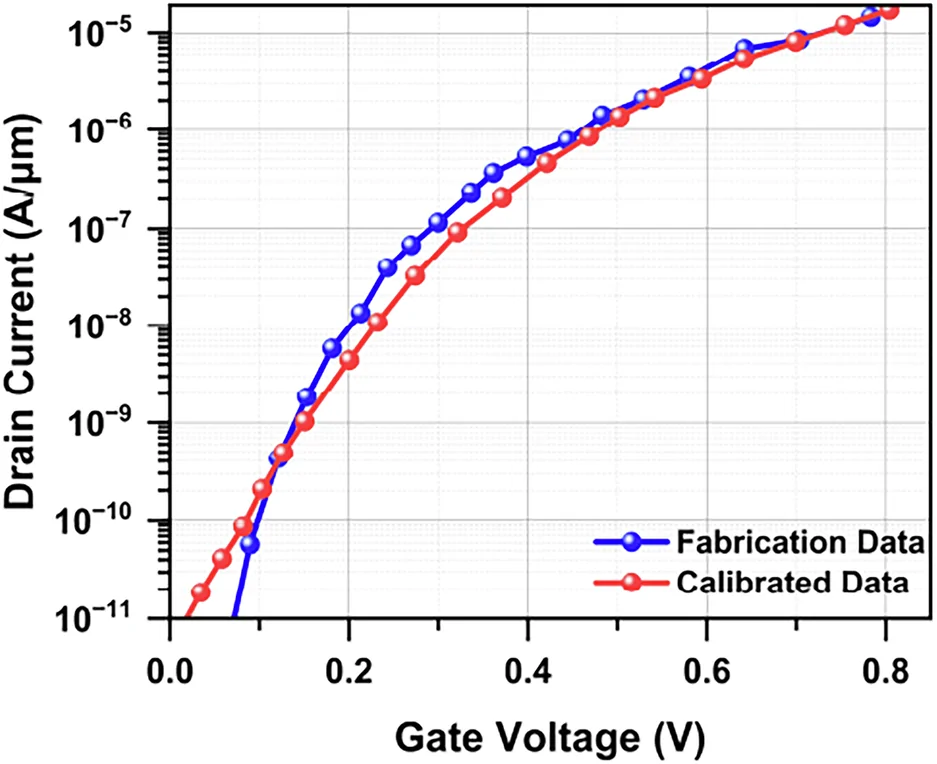
Calibration and validation of the simulated transfer characteristics against the experimental data reported in [18].

The proposed device was simulated using SILVACO TCAD to analyze its electrical characteristics. The dielectric constant, *K*, was varied to model the presence of different biomolecules. To simulate charged biomolecules, a fixed interface charge *N* is applied uniformly along the entire nano-cavity length (*L*_gap_) at the interface between the nano-cavity and the channel surface, representing the interfacial charge between the biomolecules and the channel. Six charged biomolecules are considered, with *K* = 5 and *N* = ( − 2, − 1, − 0.2, 0.2, 1, 2) × 10^12^C cm^−2^. The simulation employed the Non-Local Band-to-Band tunnelling (BBT.NONLOCAL) and Forward Bias tunnelling (BBT.FORWARD) models, with the device operating under room-temperature conditions at 300K, as (BBT.NONLOCAL) accounts the energy bands in spatial profile manner and it includes the spatial separation of electrons and holes generated in conduction and valance bands respectively [26]. The Fermi-Dirac distribution model (FERMI) was used to account for Fermi statistics in determining the intrinsic carrier concentration. Additionally, several physical models were incorporated to ensure realistic device behavior. The Shockley–Read–Hall model (SRH) was used to account for trap-assisted carrier generation and recombination via defect states, while the Auger model (AUGER) was included to capture non-radiative recombination under high carrier concentration. The transverse field-dependent mobility model (CVT) was employed to describe mobility degradation under strong normal electric fields, whereas the field-dependent mobility model (FLDMOB) accounts for carrier velocity degradation at high electric fields. The concentration-dependent mobility model (CONMOB) and concentration-dependent SRH lifetime model (CONSRH) were used to include the effects of doping on carrier mobility and lifetime, respectively. Finally, the band-gap narrowing model (BGN) was enabled to account for the effective reduction of band gap in heavily doped regions. This model set is adapted from established practice already used in previously published TFET and dielectric-modulated TFET/MOSFET biosensor simulation studies [5, 15, 16, 26] and then calibrated and validated against the experimental data reported in [18], as shown in Fig. 3. Since this reference calibration was obtained for the closely related, InP-lattice-matched In_0.53_Ga_0.47_As/GaAs_0.49_Sb_0.51_ composition, the calibration parameters like tunnelling effective masses of our own In_0.3_Ga_0.7_As/GaAs_0.75_Sb_0.25_ layers were obtained independently via Vegard-law interpolation between the respective binary end-point compounds.

## Results and discussion

**Fig. 4:**
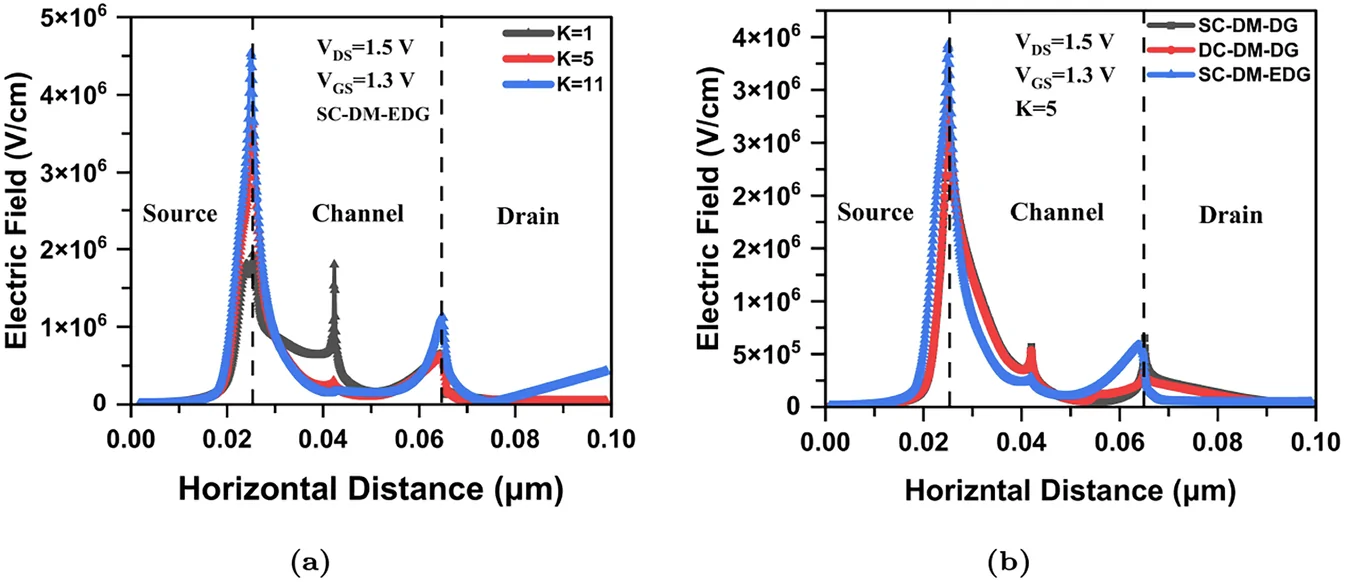
Electric field: (**a**) for different *K* values (**b**) different structures

The drain-to-source voltage *V*_DS_ is fixed at 1.5 V, while the gate-to-source voltage *V*_GS_ is varied from 0 V to 1.3 V. The introduction of biomolecules into the nano-gap, accompanied by an increase in their *K*, generates an electric field (*E*) at the source-channel tunnelling interface, as illustrated in Fig. 4a. Since the *K* increases, the coupling between gate and channel also rises dramatically, increasing the *E* in the source-channel junction, as depicted by Fig. 4a. Again, among three types of structures, the SC-DM-EDG HTFET exhibits the highest *E* illustrated in Fig. 4b for a specific *K*.

**Fig. 5:**
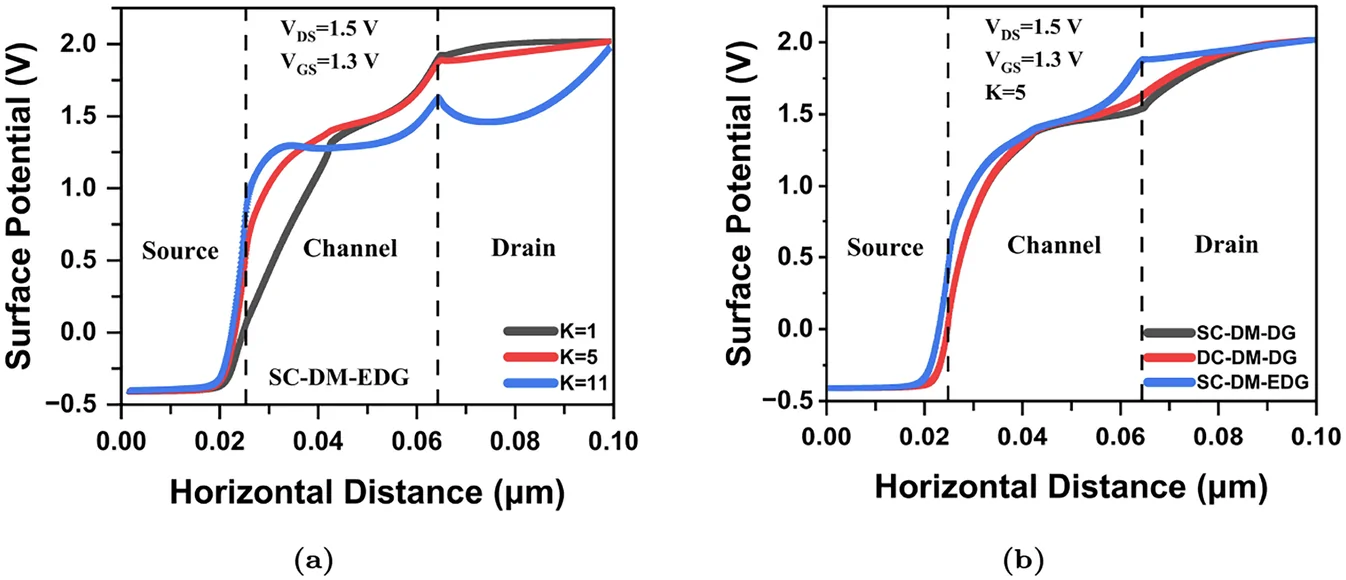
Surface potential profiles: (**a**) for different *K* values (**b**) different structures

The surface potential of the three structures is also illustrated in Fig. 5a. The gradient of surface potential is steepest for the SC-DM-EDG HTFET structure, confirming that this structure generates an enormous *E* at the source-drain junction. The surface potential for different *K* values in the SC-DM-EDG HTFET is also plotted in Fig. 5b. As *K* increases, the slope of the potential becomes steeper, confirming higher coupling between the gate and the channel.

**Fig. 6:**
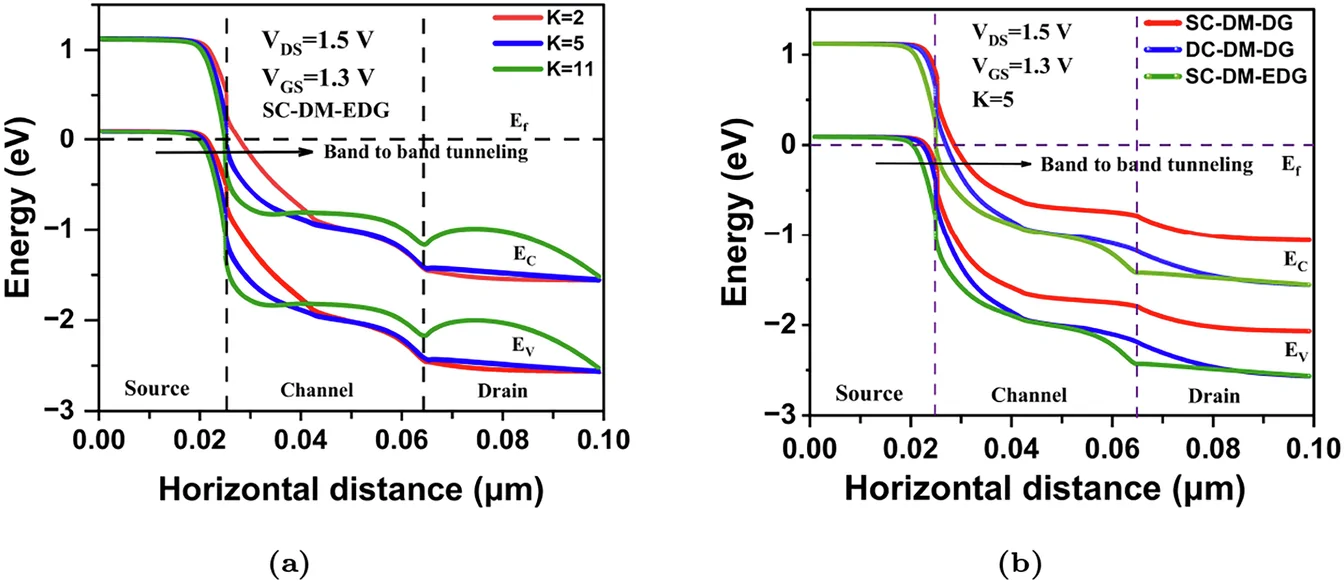
Band diagram: (**a**) for different *K* values (**b**) different structures

The conduction band edge (*E*_*C*_), valence band edge (*E*_*V*_), and overall band diagram for different dielectrics in the SC-DM-EDG HTFET are shown in Fig. 6a. As depicted in the figure, as the value of *K* increases, the band bending in the source-channel junction becomes steeper and the tunnelling length decreases. In Fig. 6b, the band diagram for different structures is shown. Overall, the band-to-band electron generation rate (*G*_BTBT_) is given by:

1 $${G}_{{\rm{BTBT}}}=A{E}^{\sigma }\exp \left(-\dfrac{B}{E}\right)$$

where *E* is the electric field, *A* is related to the effective mass of the electron, *B* is associated with the tunnelling probability, and *σ* represents the transition constant [16]. To evaluate the tunnelling probability in TFET devices, the Wentzel-Kramers-Brillouin (WKB) method is employed [27, 28], where the tunnelling probability (*T*_WKB_) is expressed as:

2 $${T}_{{\rm{WKB}}}\approx \exp \left(\dfrac{-4\lambda \sqrt{2{m}^{* }}\sqrt{{E}_{g}^{3}}}{3q\hslash ({E}_{g}+{\rm{\Delta }}\phi )}\right)$$

here, *λ* signifies the tunnelling width, m^*^ corresponds to the electron effective mass, *E*_*g*_ denotes the bandgap energy, and ${\Delta}{\phi}$ refers to the energy range across which tunnelling can occur. From Eq. (1), it can be inferred that as the dielectric constant of biomolecules within the nano-gap increases, the band bending becomes steeper, which in turn creates a stronger *E*, and consequently, the generation rate of tunnelling electrons increases significantly. From Eq. (2), it follows that an increase in the dielectric constant of biomolecules leads to a reduction in *λ*, resulting in a higher *T*_WKB_. As *T*_WKB_ increases, *I*_*ON*_ increases as well. Based on the formulas and the results presented in Figs. 4 and 6, the SC-DM-EDG HTFET is expected to exhibit the maximum *I*_*ON*_, reaching its highest value at the largest *K*, since it has the highest band bending, strongest *E*, and lowest *λ* among all the topologies.

**Fig. 7:**
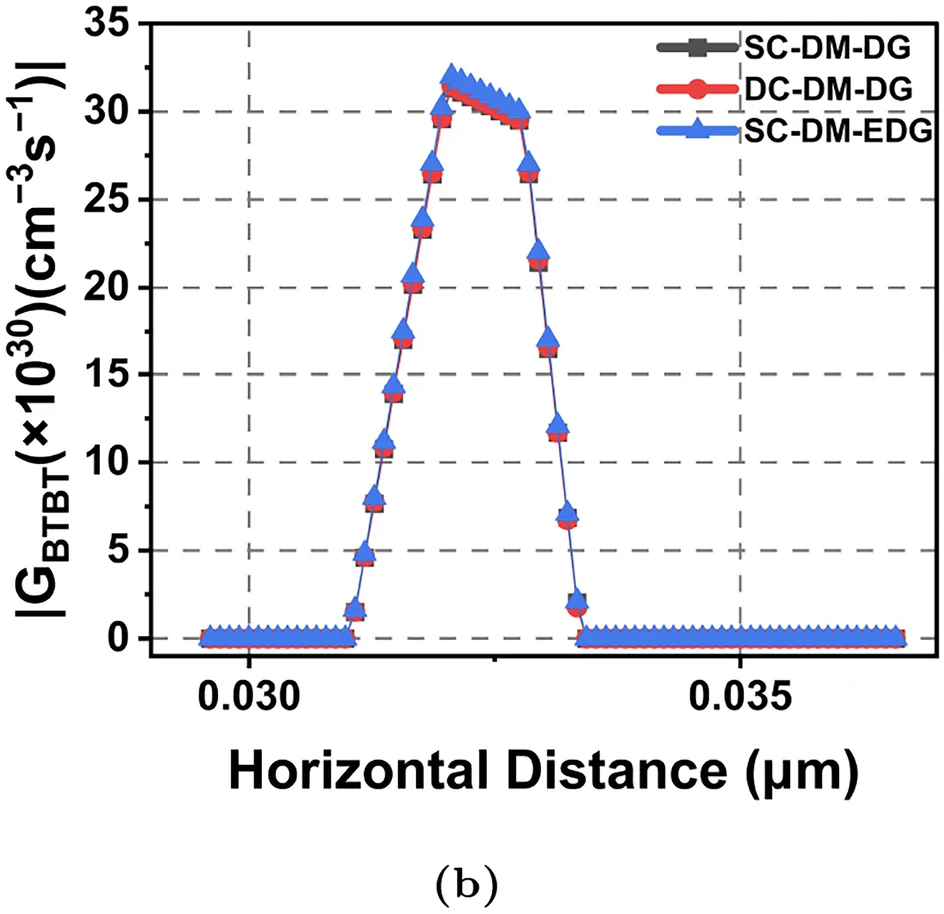
Tunnelling rate for different structures

Implementing the non-local BTBT model in SILVACO ATLAS TCAD allows the BTBT phenomenon to be addressed as a classical generation mechanism. When a biomolecule is located within the nano-gap, the electron’s contribution to current generation is significantly higher than the hole’s contribution in the NTFET, indicating a markedly higher BTBT electron generation rate. The overall non-local tunnelling rate is illustrated in Fig. 7 for the three structures. Among them, the SC-DM-EDG HTFET has the highest non-local tunnelling rate in the channel region, as expected from the analysis of Eqs. (1) and (2).

**Fig. 8:**
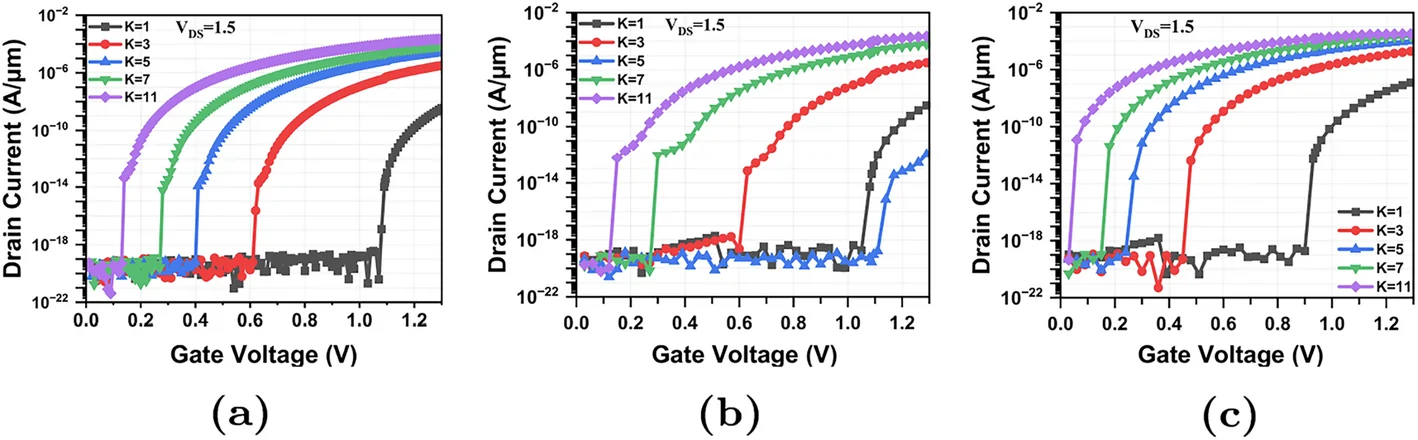
Transfer curves: (**a**) SC-DM-DG HTFET, (**b**) DC-DM-DG HTFET, (**c**) SC-DM-EDG HTFET

The DC transfer characteristics for the three structures are displayed in Fig. 8 for representative *K* values. As expected, the SC-DM-EDG HTFET has the highest *I*_*ON*_ for a specific value of *K*. *I*_*ON*_ is lowest when the nano-cavity is empty. Although the SC-DM-EDG structure has the highest *I*_*ON*_, the off current is also considerably higher compared to the other two structures under this biasing condition. That’s why, under this DC condition, the SC-DM-EDG topology will dissipate more power in the off condition. However, the *I*_*OFF*_ in these structures is in the range of 10^−18^ to 10^−21^, which is still far lower than that of dual-gate MOSFETs. A lower *I*_*OFF*_ leads to reduced static power dissipation in the TFET biosensors compared to MOSFET counterparts; however, this alone does not guarantee superior biosensor performance, since detection capability also depends on the magnitude of the *I*_*ON*_ signal change upon biomolecule binding, noise, achievable dynamic range, and the sensitivity of the readout circuitry. The current begins to conduct at a lower gate voltage in the SC-DM-EDG HTFET compared to other structures, indicating that it possesses a lower threshold voltage (*V*_*th*_). Furthermore, the current exhibits saturation with increasing *V*_GS_ beyond 1.3V, rather than pinch-off as in a conventional MOSFET.

### Sensitivity analysis

Three parameters, i.e., *g*_*m*_, *I*_*ON*_, and *V*_*th*_, from all three structures are extracted for each biomolecule with (*K* = 1, 2, 3, 5, 7, 11, 12), corresponding to air (baseline), streptavidin, biotin, gluten, zenin, keratin, and gelatin, respectively. Gelatin (*K* = 12) is treated as the representative high-K case because it is the largest dielectric constant among the commonly reported receptor/target biomolecules in this range [5, 15], allowing the device’s sensitivity and selectivity trends to be evaluated across the full span from near-unity (air) to this upper bound. Sensitivity is then calculated using the following formula [15, 29]:

3 $${S}_{{\rm{on}}}=\left\vert \dfrac{{I}_{{\rm{ON}}}({\rm{biomolecule}})}{{I}_{{\rm{ON}}}({\rm{air}})}\right\vert .$$

**Fig. 9:**
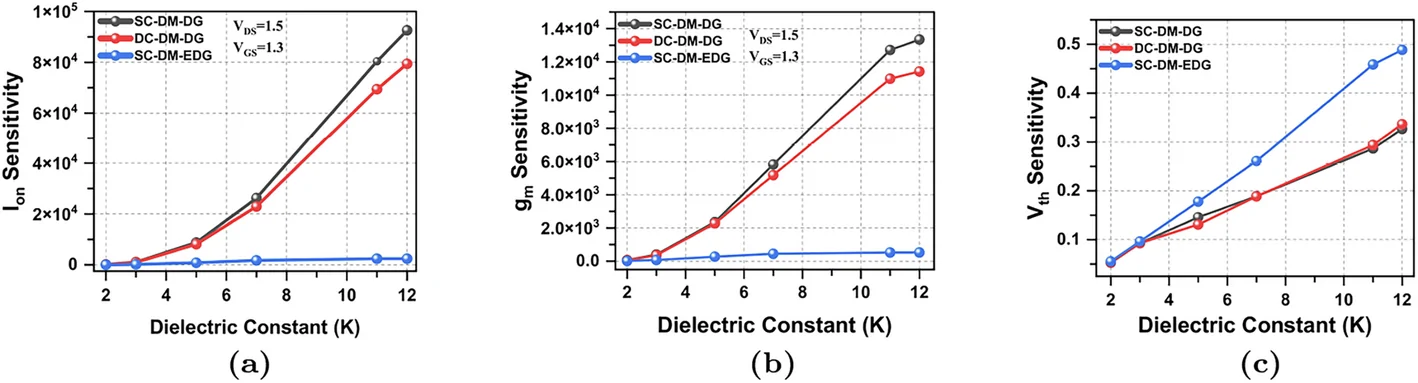
Sensitivities for different structures: (**a**) *I*_*ON*_ sensitivity, (**b**) *g*_*m*_ sensitivity, (**c**) *V*_*th*_ sensitivity

From Eq. (3), the sensitivity of *I*_*ON*_ is evaluated by the relative ratio of *I*_*ON*_ in the presence and absence of biomolecules inside the nano-gap and illustrated in Fig. 9a. Similarly, *g*_*m*_ sensitivity is defined by taking the ratio between *g*_*m*_ at *K* = 1 (air) and *g*_*m*_ in the presence of biomolecules. Finally, *V*_*th*_ sensitivity is calculated using the difference between *V*_*th*_ with specific biomolecules filling the nano-gap and *V*_*th*_ when the nano-gap is empty. *g*_*m*_ and *V*_*th*_ sensitivities are illustrated in Figs. 9b and c, respectively. Figure 9a exhibits the variation of *I*_*ON*_ sensitivity with *K* for the three biosensor structures. It is observed that the sensitivity increases with higher *K* due to stronger gate-to-channel coupling, which enhances the *E* at the source-channel tunnelling junction, thereby reducing tunnelling width and increasing tunnelling probability. Among the devices, the SC-DM-DG structure exhibits the highest sensitivity, followed by DC-DM-DG. Interestingly, although the SC-DM-EDG HTFET demonstrates the maximum absolute *I*_*ON*_ owing to its strong electric field and steep surface potential profile, its sensitivity remains lower. This is attributed to the relatively high baseline current in the absence of biomolecules, which reduces the relative change upon biomolecule introduction. Consequently, under the applied bias conditions (*V*_DS_ = 1.5), the SC-DM-EDG structure already achieves enough band alignment to flow current when there is air in the nano-cavity. Again, as the extended gate is quite close to the tunnel junction, the electric field coupling is higher even if no biomolecule is present. So, under this high supply voltage, the SC-DM-EDG structure has a higher current drive capability at the expense of sensitivity, whereas the SC-DM-DG configuration provides superior sensitivity performance. The highest *I*_*ON*_ sensitivity is almost 1 × 10^5^, which is achieved by the SC-DM-DG HTFET structure for *K* = 12.

In a similar manner, the SC-DM-EDG HTFET shows the highest *g*_*m*_ value, although its sensitivity is quite lower due to the bias condition, as it allows current to flow even if there is no biomolecule present. The SC-DM-DG HTFET achieves the highest *g*_*m*_ sensitivity of 1.3 × 10^4^ for *K* = 12. However, the pattern is quite opposite in the case of *V*_*th*_ sensitivity. SC-DM-EDG HTFET achieves the highest sensitivity of approximately 0.5 for *K* = 12, while the figure for SC-DM-DG HTFET is only around 0.3.

Besides sensitivity, the *I*_*ON*_ is another crucial factor for biomolecule detection. If the on current is not adequately high, it may cause false detection or even complete failure of detection. Conventional MOSFET-based biosensors typically provide large *I*_*ON*_ values, whereas homojunction TFETs fall short in delivering strong on-current. In contrast, these heterojunction designs offer sufficiently high *I*_*ON*_ for detecting biomolecules with larger *K*. Among them, the SC-DM-EDG HTFET structure delivers greater on-current compared to the other two architectures, thereby simplifying the detection process.

**Fig. 10:**
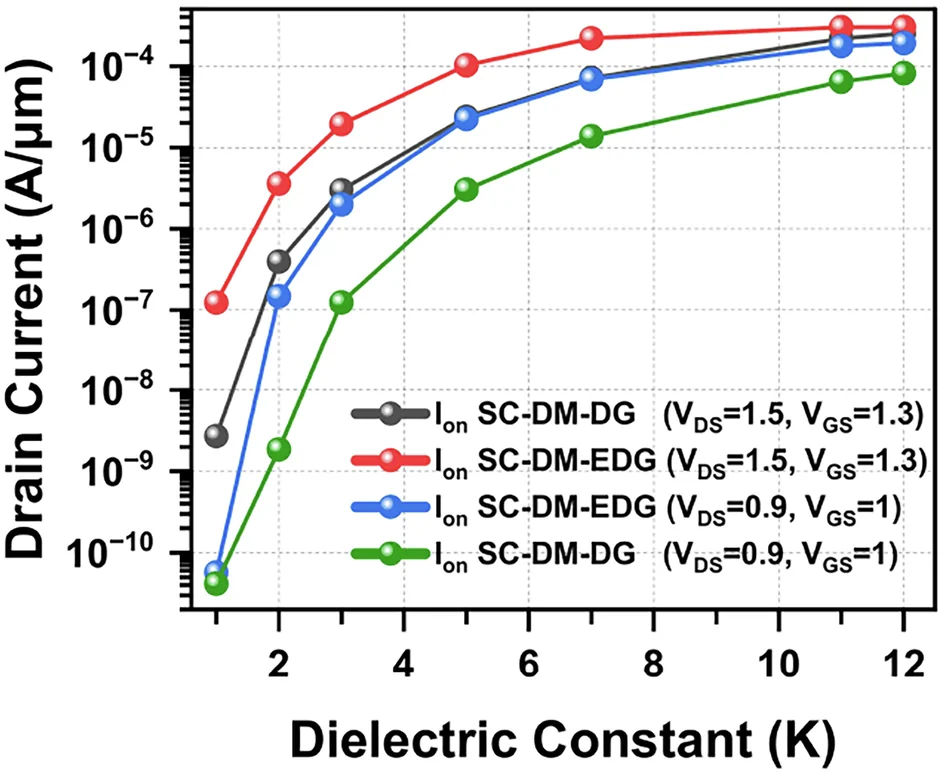
*I*_*ON*_ vs. *K* for different biasing schemes

At the applied bias (*V*_DS_ = 1.5 V, *V*_GS_ = 1.3 V), the SC-DM-EDG HTFET induces enough band bending and reduces the tunnelling distance sufficiently so that current flows even in the absence of biomolecules (*K* = 1). This explains why the sensitivity remains quite low under this DC bias point, as the current change after biomolecule introduction is not significant. That’s why, optimal bias point (*V*_DS_ = 0.9 V, *V*_GS_ = 1 V) was identified by repeatedly sweeping and recalibrating the simulation over a range of *V*_DS_ and *V*_GS_ values, rather than being fixed a priori. By adjusting the bias to this value, the sensitivity of the SC-DM-EDG HTFET improves dramatically. However, this improvement in sensitivity comes at the cost of reduced *I*_*ON*_. Still, the current achieved by the SC-DM-EDG HTFET under this condition is comparable to that of the SC-DM-DG HTFET at the earlier bias point. The overall *I*_*ON*_ variations are illustrated in Fig. 10.

As shown in Fig. 10, the *I*_*ON*_ of the SC-DM-DG HTFET under the new bias condition is relatively low, which makes biomolecule detection difficult, even though its sensitivity is comparable to that of the other two topologies. The DC-DM-DG structure shares the same non-extended gate electrostatics as SC-DM-DG and is therefore expected to show the same *I*_*ON*_ collapse under this bias condition; it was not simulated separately at this operating point. In contrast, the SC-DM-EDG HTFET delivers sufficient on-current along with improved sensitivity in this biasing scheme. Furthermore, it can be observed that in the absence of biomolecules, the current of the SC-DM-EDG HTFET is noticeably lower than the current of the SC-DM-DG HTFET under the previous biasing condition.

**Fig. 11:**
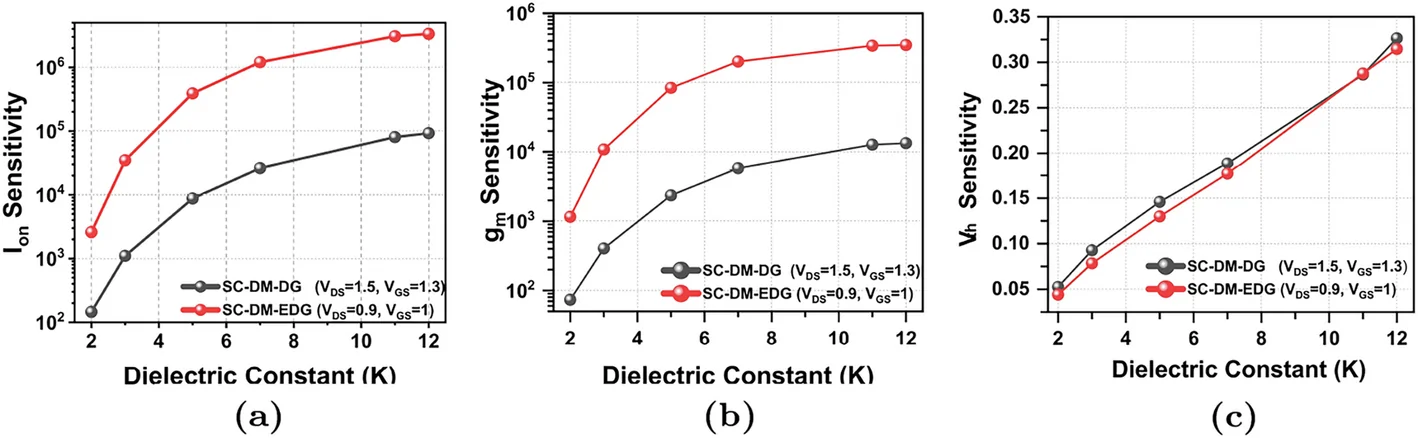
Sensitivity comparison between SC-DM-DG HTFET and SC-DM-EDG HTFET: (**a**) *I*_*ON*_ sensitivity, (**b**) *g*_*m*_ sensitivity, (**c**) *V*_*th*_ sensitivity

The sensitivities of the SC-DM-EDG TFET are illustrated in Fig. 11. Under the new biasing condition, this structure exhibits orders of magnitude higher sensitivity in both *I*_*ON*_ and *g*_*m*_ compared to the previously best-performing SC-DM-DG HTFET. This improvement highlights the superior capability of the SC-DM-EDG design in amplifying biomolecular effects within the tunnelling junction, making it a more effective candidate for high-performance biosensing applications.

### Selectivity analysis

The selectivity is defined as the ability to distinguish between biomolecules and is given by [11]:

4 $$\,\mathrm{Selectivity}\,,\ {\rm{\Delta }}S=\dfrac{{\rm{\Delta }}{I}_{{\rm{ON}}}}{{I}_{{\rm{ON}}}(K=5)}$$

where Δ*I*_ON_ = *I*_ON_(*K* = 2, 3, 7, 11, 12) ~ *I*_ON_(*K* = 5). Selectivity (Δ*S*) is a crucial figure of merit in TFET-based biosensors, as it defines the ability of the device to electrically discriminate between the dielectric constants associated with different biomolecules, each individually assumed to fully occupy the nano-cavity. As defined here, Δ*S* reflects the device’s response to differences in assumed dielectric constant rather than a demonstrated capability to selectively detect a target biomolecule amid interfering species, mixed occupancy, or non-specific binding; establishing the latter would additionally require receptor-level specificity and competitive-binding effects, which are beyond the scope of the present dielectric-constant-based model, as noted in the discussion of experimental and receptor-chemistry limitations above.

**Fig. 12:**
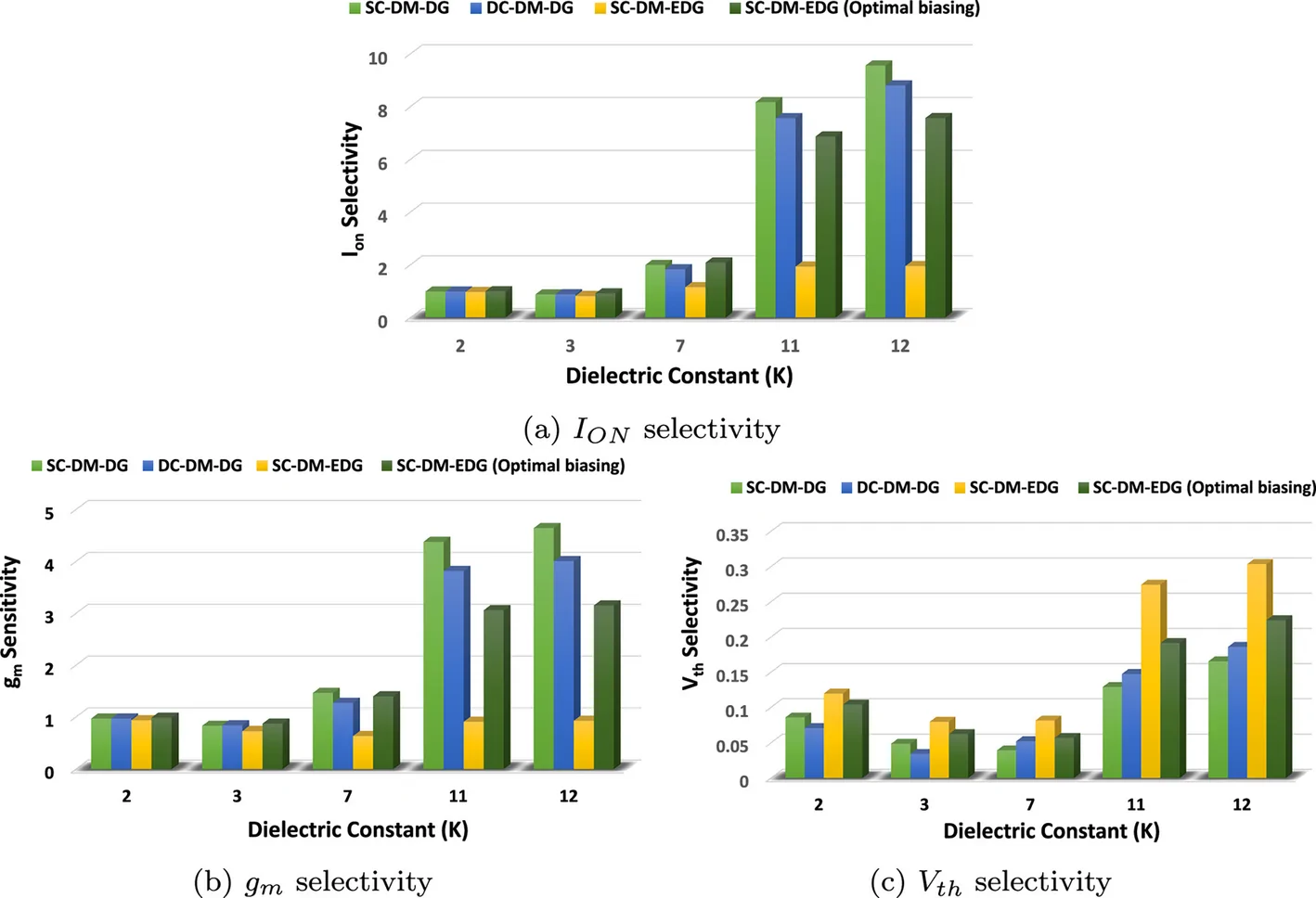
Selectivity comparison between three topologies: (**a**) *I*_*ON*_ selectivity, (**b**) *g*_*m*_ selectivity, and (**c**) *V*_*th*_ selectivity

The selectivity for all three structures is plotted in Fig. 12. Maximum *I*_*ON*_ selectivity is approximately 10, achieved by the SC-DM-DG HTFET structure for *K* = 12. Thus, the single cavity structure can distinguish between biomolecules more effectively than the other two. Similarly, *g*_*m*_ selectivity is also higher for the SC-DM-DG structure, and maximum selectivity is for *K* = 12, which is around 5. As a result, biomolecules like gelatin (*K* = 12) will be highly distinguishable for this proposed work. Finally, *V*_*th*_ selectivity is highest for the SC-DM-EDG structure, which is around 0.30, and it is also for *K* = 12.

### Impact of charged biomolecules

In addition, certain biomolecules, such as DNA, may undergo ionization once they bind with the receptors. It becomes essential to study the operation of a biosensor when both neutral and charged biomolecules are present. The voltage balance expression for a MOSFET can be written as [16]:

5 $${V}_{{\rm{GS}}}={\psi }_{{\rm{s}}}+{\phi }_{{\rm{ms}}}-\left(\dfrac{+N}{{C}_{{\rm{eff}}}}\right)$$

here, *ψ*_s_ denotes the surface potential, *N* represents the charged biomolecules, *C*_eff_ is the effective capacitance per unit area, and ${\phi}_{ms}$ is the metal-semiconductor work function. Eq. (5) indicates that, for a fixed *V*_GS_ and a constant dielectric property of biomolecules, an increase in  + *N* leads to a rise in *ψ*_s_, thereby improving the device sensitivity. This enhancement originates from the growth of the factor $\frac{+N}{{C}_{{\rm{eff}}}}$ at the nano-cavity/channel interface, which enhances electron accumulation in the lightly doped n-type InGaAs channel beneath the nano-cavity at lower gate bias. As a result, sharp band bending occurs at the source-channel junction. This process lowers the threshold voltage and raises the *I*_*ON*_ of the structure [16].

**Fig. 13:**
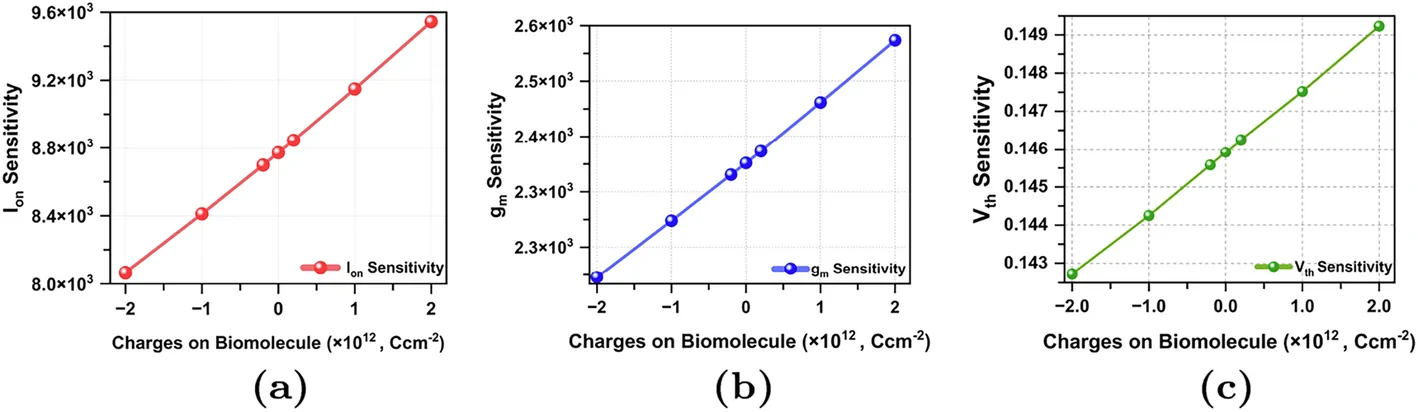
SS sensitivity vs. biomolecule charge for SC-DM-DG HTFET at *K* = 5: (**a**) *I*_*ON*_ sensitivity, (**b**) *g*_*m*_ sensitivity, (**c**) *V*_*th*_ sensitivity

**Fig. 14:**
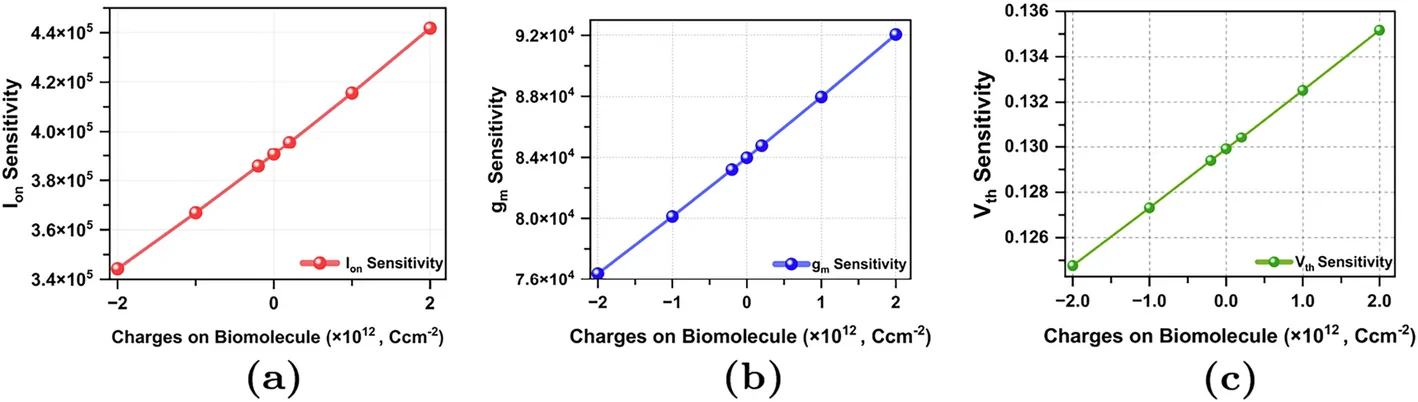
Sensitivity vs. biomolecule charge for SC-DM-EDG HTFET at *K* = 5: (**a**) *I*_*ON*_ sensitivity, (**b**) *g*_*m*_ sensitivity, (**c**) *V*_*th*_ sensitivity

On the other hand, if  − *N* increases, then *ψ*_s_ decreases, reducing device sensitivity. This occurs because of the increased $\frac{-N}{{C}_{{\rm{eff}}}}$ factor at the nano-cavity/channel interface, which reduces electron accumulation in the lightly doped n-type InGaAs channel and elevates the threshold voltage. Consequently, the tunnelling width at the source-channel interface grows, which suppresses the tunnelling rate, and thus decreases the *I*_*ON*_ of the biosensor. In this work, the biomolecule charge *N* is varied from *N* = − 2 × 10^12^ C cm^−2^ to *N* = + 2 × 10^12^ C cm^−2^. The overall variation of sensitivities with respect to the charge, keeping *K* at a constant value of 5, is shown in Figs. 13 and 14 for SC-DM-DG and SC-DM-EDG topologies, respectively. As evident from the figures, the sensitivities increase if positive charge is introduced, and the opposite happens in the case of negative charges, as expected from Eq. (5).

**Fig. 15:**
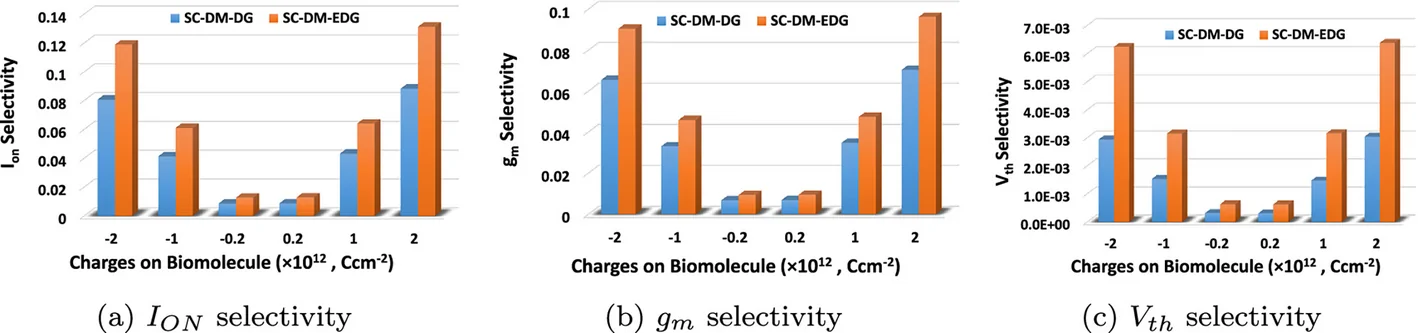
Selectivity comparison between SC-DM-DG and SC-DM-EDG for charged biomolecules: (**a**) *I*_*ON*_ selectivity, (**b**) *g*_*m*_ selectivity, (**c**) *V*_*th*_ selectivity

In Fig. 15, the variation of selectivity is shown. It is quite apparent from the graph that the SC-DM-EDG topology shows higher selectivity in *I*_*ON*_, *g*_*m*_, and *V*_*th*_ compared to the SC-DM-DG structure. This means SC-DM-EDG is superior in detecting and differentiating charged molecules from neutral ones. All performance parameters of the simulated topologies are summarized in Table 1.

**Table 1:** Peak Metric values for different HTFET structures

Metric	SC-DM-DG	DC-DM-DG	SC-DM-EDG	SC-DM-EDG
	HTFET	HTFET	HTFET	HTFET
				(Optimal Biasing)
*I*_*ON*_ (A/*μ*m)	2.51 × 10^−4^	2.15 × 10^−4^	3.02 × 10^−4^	1.91 × 10^−4^
*I*_*ON*_ Sensitivity	9.26 × 10^4^	7.94 × 10^4^	2.43 × 10^3^	3.35 × 10^6^
*g*_*m*_ Sensitivity	1.33 × 10^4^	1.14 × 10^4^	5.31 × 10^2^	3.49 × 10^5^
*V*_*th*_ Sensitivity	0.33	0.34	0.49	0.31
*I*_*ON*_ Selectivity	9.55	8.80	1.95	7.57
*g*_*m*_ Selectivity	4.64	4.00	0.93	3.15
*V*_*th*_ Selectivity	0.17	0.19	0.30	0.22

### Noise and practical detection considerations

The reported sensitivity and selectivity are based on deterministic DC characteristics, whereas practical TFET biosensor operation is also affected by noise, variability, and non-ideal cavity filling. A quantitative noise figure of merit for FET biosensors is ${\rm{SNR}}=({\rm{\Delta }}{\psi }_{0}/\sqrt{BW})\times [{g}_{m}/\sqrt{{S}_{I}}]$, where Δ*ψ*_0_ is the biomolecule-induced surface-potential shift, *g*_*m*_ the transconductance, *S*_*I*_ the current noise power spectral density, and *BW* the measurement bandwidth [30]. Evaluating it, and the corresponding detection limit, requires noise-aware simulation (flicker, shot, and random-telegraph-noise modelling) or measured device data, neither available within the present deterministic TCAD framework. III–V heterojunction TFETs reportedly show lower flicker-noise impact than homojunction devices, with low-frequency noise dominated by trap states near the tunnelling junction rather than the channel-mobility fluctuations governing MOSFET noise [31], suggesting favorable noise behavior for this device family, though not separately verified for the GaAsSb/InGaAs HTFETs studied here. A quantitative SNR, detection limit, and noise margin remain a direction for future noise-aware simulation and experimental work. Moreover, steric hindrance caused by receptor/linker molecules and partial filling of the nanocavity can weaken dielectric modulation by reducing the effective biomolecule occupancy. As demonstrated in [5], such effects may be modeled more completely in future work using different cavity-filling profiles to emulate incomplete or nonuniform biomolecule immobilization. As a limited check, we approximated fractional gelatin (*K* = 12) cavity occupancy using an effective dielectric constant *K*_*eff*_ = *fK* + (1 − *f*)*K*_*air*_, where *f* ∈ [0, 1] is the occupied nano-cavity fraction and *K*_*air*_ = 1, for SC-DM-EDG topology under re-optimized bias (*V*_*DS*_ = 0.9 V, *V*_*GS*_ = 1 V). The *I*_*ON*_ sensitivity falls from 3.35 × 10^6^ at full occupancy to roughly 2.0 × 10^6^ at 75% and 8.7 × 10^5^ at 50% occupancy, then drops sharply to  ~ 7.7 × 10^4^ at 25% occupancy. The same qualitative trend (moderate partial filling has limited impact; low occupancy substantially degrades sensitivity) seen at standard bias condition (*V*_*DS*_ = 1.5 V, *V*_*GS*_ = 1.3 V) as well. A full quantitative treatment of interface traps, trap-assisted tunnelling, and process-induced variability remains beyond the present study’s scope and is left for future experimental validation. Even so, the strong current and threshold-voltage modulation predicted here still suggests promising intrinsic sensing potential.

Several additional factors relevant to experimental realization remain outside this simulation study’s scope. The proposed fabrication route (Fig. 2) is adapted from demonstrated GaAsSb/InGaAs heterojunction diode processing [18], but integrating the extended dual-material gate and nanocavity at the stated dimensions has not been experimentally verified and may be affected by process variation in gate length, cavity height, and doping. Interface trap states at the gate-dielectric/channel and nano-cavity/channel interfaces, and random dopant fluctuations at the heavily doped source-channel junction, are additional expected sources of device-to-device variability in *I*_*OFF*_ and *I*_*ON*_ not captured by the present deterministic TCAD framework, requiring statistical or atomistic-level simulation to quantify. Because band-to-band tunnelling is inherently less thermally activated than the thermionic emission governing MOSFET operation, TFETs are generally reported to show weaker temperature dependence of subthreshold swing and on-current than MOSFETs; this expected trend has not been separately verified here and is left for future electro-thermal characterization. The simulations also omit electrolyte screening: in a physiological buffer, mobile ions can screen the biomolecule-induced field over the Debye length, potentially reducing the sensitivity reported here for charged-biomolecule detection (Fig. 13 and 14). Receptor chemistry linker length, surface density, and binding orientation was likewise not modeled, though it can affect both the effective dielectric constant and achievable cavity occupancy; experimental fabrication and measurement of the proposed device are needed to validate the predicted performance. Finally, the dielectric constants used here, including gelatin’s *K* = 12, are each biomolecule’s commonly reported effective/dry-state value assuming uniform nanocavity occupancy; hydration and physiological-buffer effects on the effective permittivity are not modeled explicitly, consistent with the convention adopted in prior dielectric-modulated TFET biosensor studies [5, 6].

### Disentangling the sources of sensitivity enhancement

Because three architectural changes are examined here (cavity placement, gate extension, and bias point), it is necessary to isolate which one is responsible for the reported sensitivity gain, rather than crediting the combined architecture in general terms. Table 1 makes this decomposition explicit. At matched bias (*V*_DS_ = 1.5 V, *V*_GS_ = 1.3 V), changing only the cavity configuration (SC-DM-DG  → DC-DM-DG) slightly *reducesI*_*ON*_ sensitivity (9.26 × 10^4^ → 7.94 × 10^4^), showing that TFET sensing performance is governed by the source-channel tunnelling junction, not by cavity placement. Adding the extended gate at the same bias (SC-DM-DG  → SC-DM-EDG) raises *I*_*ON*_ (2.51 × 10^−4^ → 3.02 × 10^−4^ A/*μ*m) but *reduces* sensitivity by nearly two orders of magnitude, to 2.43 × 10^3^, because the stronger junction coupling sustains measurable current even with air (*K* = 1) in the cavity, inflating the baseline used to normalize Eq. (3). Neither change alone, therefore, explains the highest reported sensitivity: 3.35 × 10^6^ is obtained only when the extended gate is combined with a re-optimized bias (*V*_DS_ = 0.9 V, *V*_GS_ = 1 V) that suppresses the air-cavity baseline while keeping *I*_*ON*_ (1.91 × 10^−4^ A/*μ*m) at the same order as the other structures. This bias shift affects selectivity differently: *I*_*ON*_ selectivity falls from 9.55 to 1.95 at matched bias and only partially recovers, to 7.57, remaining below the original SC-DM-DG value unlike sensitivity, which rises to roughly 36 × the SC-DM-DG baseline. Sensitivity and selectivity are therefore not simultaneously maximized; the re-optimized bias point is best understood as a sensitivity-selectivity-current trade-off, not a uniform improvement. The corresponding air-cavity (*K* = 1) currents, *I*_*ON*_(*K* = 12)/*S*_*on*_, are 2.71 × 10^−9^ A/*μ*m (SC-DM-DG), 2.71 × 10^−9^ A/*μ*m (DC-DM-DG), 1.24 × 10^−7^ A/*μ*m (SC-DM-EDG, standard bias), and 5.70 × 10^−11^ A/*μ*m (SC-DM-EDG, optimal bias) confirming Δ*I*_*ON*_ stays comparable ( ~ 10^−4^ A/*μ*m) across all four cases. As shown in Fig. 10, applying the same bias shift to SC-DM-DG does not reproduce this outcome: its *I*_*ON*_ collapses to an impractically low level, and DC-DM-DG, sharing the same non-extended-gate electrostatics, is expected to behave the same way, since cavity placement was already shown to have only a marginal effect on sensitivity. The extended gate is therefore not a direct source of higher sensitivity but the architectural feature that makes this *I*_*ON*_-sensitivity trade-off exploitable at all distinguishing this work from prior GaAsSb/InGaAs dual-material-gate biosensors [22, 23], which report sensitivities of 10^4^–10^5^ without examining gate extension or bias re-optimization, and from prior extended-gate biosensors on IV–IV or InGaAs/Si platforms [5], which do not combine gate extension with a type-II GaAsSb/InGaAs junction or cavity-placement trade-offs.

## Comparison with other work

The comparative analysis in Table 2 highlights that conventional and early TFET biosensors offered limited sensitivity, typically ranging from 10 to 10^2^, due to weak electrostatic control and high tunnelling barriers. Later architectures, including double-gate and dual-metal designs, enhanced gate coupling and achieved sensitivities up to 10^5^-10^6^[32–34]. The proposed GaAsSb/InGaAs SC-DM-EDG HTFET achieves a sensitivity of 3.35 × 10^6^ under a re-optimized bias point (at a correspondingly reduced *I*_ON_ of 1.91 × 10^−4^ A/*μ*m), while, as shown in Disentangling the Sources of Sensitivity Enhancement section, the same structure separately delivers the highest *I*_ON_ of 3.02 × 10^−4^ A/*μ*m among all three architectures under the standard bias condition used for the sensitivity and selectivity comparisons in Figs. 9 and 12. This improvement stems from the type-II heterojunction that enhances BTBT, combined with an extended dual-material gate (EDG) that strengthens gate control and electric field distribution and, in doing so, makes the two operating points (maximum current drive and maximum sensitivity) simultaneously accessible from a single geometry via bias selection alone—a flexibility that Fig. 10 shows is not available to the same degree in the non-extended architectures. Furthermore, the single-cavity structure ensures simplified fabrication while maintaining high performance, making the proposed SC-DM-EDG HTFET a promising candidate for low-power, ultra-sensitive biosensing applications compared to existing TFET and MOSFET-based biosensors.

**Table 2:** Comparison of the proposed device with previously reported dielectric-modulated nanocavity MOSFET and TFET biosensors

Device/Structure	Ref	Sensitivity
Lateral DM TFET (*L*_gap1_ = 10 nm, *H*_gap_ = 5 nm, *K* = 2)	[35]	10
Double gate TFET (*L*_gate_ = 10 nm, *L*_gap_ = 10 nm, *H*_gap_ = 5 nm, *K* = 5)	[34]	25
Vertical DM TFET (*L*_gap1_ = 10 nm, *L*_gap2_ = 15 nm, *H*_gap_ = 5 nm, *K* = 2)	[36]	40
Conventional TFET	[37]	50
Double gate (DG) TFET (*L*_gap_ = 30 nm, *L*_gate_ = 40 nm, *K* = 12)	[32]	1.05 × 10^2^
SiGe Source DM PNPN TFET with 10% Ge composition	[14]	6 × 10^3^
SiGe source DM PNPN TFET with 10% Ge composition (*L*_gap_ = 15 nm, *L*_gate_ = 100 nm, *H*_gap_ = 9 nm, *K* = 2.1)	[38]	6 × 10^3^
C–DMDG TFET (*L*_gap_ = 10 nm, *L*_M1_ = *L*_M2_ = 15 nm, *H*_gap_ = 5 nm, *K* = 12)	[33]	1.59 × 10^4^
n^+^ pocket–doped Vertical TFET (*L*_gate_ = 30 nm, *H*_gap_ = 5 nm, *L*_gap_ = 10 nm, *K* = 5)	[34]	2 × 10^4^
Buried strained Si_1−*x*_Ge_*x*_ DGTFET (*L*_gap_ = 20 nm, *L*_gate_ = 40 nm, *H*_gap_ = 5 nm, *K* = 8)	[13]	2.6 × 10^5^
N^+^ pocket–doped vertical TFET (*L*_gap_ = 20 nm & 30 nm, *L*_gate_ = 40 nm & 30 nm, *K* = 10)	[32]	3 × 10^5^
GaAs/InAs DMDG–TFET (*L*_gap_ = 10 nm, *L*_m1_ = *L*_m2_ = 15 nm, *H*_gap_ = 5 nm, *K* = 12)	[33]	6.11 × 10^5^
Dual Metal Gate SiGe pocket Vertical TFET (*L*_gap1_ = 20 nm, *L*_gap2_ = 10 nm, *H*_gap_ = 5 nm, *L*_gate1_ = 25 nm, *L*_gate2_ = 15 nm, *K* = 12)	[34]	2 × 10^6^
Dual Material Double Gate GaAsSb/InGaAs HTFET	[22]	1.56 × 10^5^
This Work		
DC–DM–DG HTFET (*L*_gate1_ = 10 nm, *L*_gate2_ = 30 nm, *L*_gap1_ = 17 nm, *L*_gap2_ = 10 nm, *H*_gap_ = 6 nm)	—	7.94 × 10^4^
SC–DM–DG HTFET (*L*_gate1_ = 10 nm, *L*_gate2_ = 30 nm, *L*_gap_ = 17 nm, *H*_gap_ = 6 nm)	—	9.26 × 10^4^
SC–DM–EDG HTFET (*L*_gate1_ = 10 nm, *L*_gate2_ = 30 nm, *L*_EG_ = 6 nm, *L*_gap_ = 17 nm, *H*_gap_ = 6 nm), re-optimized bias (*V*_DS_ = 0.9 V, *V*_GS_ = 1 V)	—	3.35 × 10^6^

## Conclusion

A GaAsSb/InGaAs-based SC-DM-EDG HTFET nanocavity biosensor has been proposed and analyzed for ultra-sensitive detection of neutral and charged biomolecules. Leveraging a type-II heterojunction and an extended dual-material gate, the device enhances tunnelling efficiency and electric field coupling at the source-channel interface, achieving a sensitivity of 3.35 × 10^6^ (*V*_DS_ = 0.9 V, *V*_GS_ = 1 V) and a high *I*_*ON*_ of 3.02 × 10^−4^ A/*μ*m (standard bias, *V*_DS_ = 1.5 V, *V*_GS_ = 1.3 V), among the highest reported for GaAsSb/InGaAs-based TFET biosensors. The single-cavity design ensures strong dielectric modulation while simplifying fabrication, and flexible biasing enables operation for either maximum sensitivity or peak current drive. These features, combined with potential material-level optimizations such as high-*K* dielectrics, tailored gate work functions, and channel engineering, make the SC-DM-EDG HTFET a promising candidate for integration into lab-on-chip and point-of-care biosensing platforms, advancing high-performance, low-power nanoelectronic biosensors.

## Data Availability

Data will be made available upon request from corresponding author.
